# Letter to the editor: Occurrence of modified measles during outbreak in Taiwan in 2018

**DOI:** 10.2807/1560-7917.ES.2018.23.37.1800485

**Published:** 2018-09-13

**Authors:** Chih-Jung Chen, Tzou-Yien Lin, Yhu-Chering Huang

**Affiliations:** 1 Division of Pediatric Infectious Diseases, Department of Pediatrics, Chang Gung Memorial Hospital, Taoyuan, Taiwan; 2 School of medicine, College of Medicine, Chang Gung University, Taoyuan, Taiwan

**Keywords:** measles, Taiwan, seroprevalence

**To the editor:** We read with great interest the rapid communication by Mizumoto et al. [1] regarding modified measles in Japan, 2018. In particular, we discovered that the index case in Japan was epidemiologically linked to an outbreak in Taiwan [2]; measles was transmitted on board an aircraft while the case returned to Taiwan from Thailand, before travelling on to Japan while febrile and contagious. Measles was confirmed in three members of the cabin crew and one passenger that shared the same aircraft as the index case. Further transmission by the infected cabin crew members occurred and involved an additional seven staff. One passenger that contracted measles in the airport caused a secondary cluster of four cases with ca 1,000 contacts in Chang Gung Memorial Hospital, Taiwan.

The first case of the hospital cluster had a delay in diagnosis, as they presented with a modified clinical picture of measles, with only fever for the first 5 days before developing rash and red eyes. Additional fully vaccinated cases presented, also without the full clinical symptoms of measles; two presented with fever initially, developing rash a few days later, but had no cough, coryza or conjunctivitis during the illness. In another case, only a generalised rash presented, while they had no fever during the course of illness.

In 2018, during the outbreak in Taiwan, from March to April involving 24 cases [3], we found that in the aircraft cluster 12 of 13 measles cases were young adults aged 20–40 years who had been immunised with two doses of measles-containing vaccine during childhood [4]. The presentation of measles was modified in these cases, making the clinical suspicion of measles very difficult; in the serological tests, measles IgM was mostly negative and the laboratory diagnosis was largely dependent on polymerase chain reaction (PCR). In cases presenting with modified measles transmission was limited, as further infections occurred in only three cases among 1,000 contacts in the hospital cluster and these cases had close and long-time contact with the modified measles cases.

Although the uptake of two-dose measles vaccine in children was maintained at > 95% for 40 years in Taiwan, the measles outbreak in younger age groups was not unexpected. We have shown that vaccine-induced humoral immunity to measles can wane to a very low level (50–60%) in young adults [4]. The low seroprevalence in young and middle-aged adults was also observed in the investigation of our hospital cluster. In this cluster, although in a much smaller sample, the seropositivity rates for anti-measles IgG among healthcare workers with contact to the modified measles cases, was only 59.7% (n = 34/57; 95% confidence interval (CI): 46.9‒72.4) and 66.7% (n = 18/27; 95% CI: 48.9‒84.5) in those aged 21–30 and 31–40 years, respectively. In contrast, the rate was 97.6% (n = 120/123; 95% CI: 94.8‒100) in contacts aged > 40 years of age who have natural immunity from contracting measles in childhood. Cellular immunity, which is an essential component against measles in addition to anti-measles IgG, was not evaluated during the epidemic. Nevertheless, our observation on the features of the infected cases strongly suggested the waning vaccine-induced humoral immunity did have consequence in the disease control. The measles outbreak in Taiwan seems to have mainly resulted from secondary vaccine failure rather than suboptimal vaccination coverage. In response to the outbreak, an additional dose was recommended to all healthcare workers and staff involved in the air transportation industry in Taiwan. Further, we also suggested a booster dose for young adults who plan to travel to areas with circulating measles. In the future, a booster dose during adulthood to enhance population immunity may be necessary to prevent further outbreaks, even in settings with high-vaccine coverage such as in Taiwan.
